# Clinical characteristics and treatment outcomes in acromegaly, a retrospective single-center case series from Thailand

**DOI:** 10.11604/pamj.2021.40.31.29920

**Published:** 2021-09-10

**Authors:** Poranee Ganokroj, Sarat Sunthornyothin, Rungsak Siwanuwatn, Kraisri Chantra, Patinut Buranasupkajorn, Sompongse Suwanwalaikorn, Thiti Snabboon

**Affiliations:** 1 Department of Laboratory Medicine, Faculty of Medicine, Chulalongkorn University, Bangkok, Thailand,; 2 Division of Endocrinology and Metabolism, Department of Medicine, Faculty of Medicine, Chulalongkorn University, Bangkok, Thailand,; 3 Excellence Center in Diabetes, Hormone and Metabolism, King Chulalongkorn Memorial Hospital, Thai Red Cross Society, Bangkok, Thailand,; 4 Division of Neurosurgery, Department of Surgery, Faculty of Medicine, Chulalongkorn University, Bangkok, Thailand

**Keywords:** Acromegaly, gigantism, pituitary tumor

## Abstract

**Introduction:**

acromegaly, an overproduction of growth hormone (GH), is associated with high rate of morbidity and mortality particularly in case of delayed in diagnosis and treatment. A wide variation of clinical presentations, treatment outcomes and morbidities have been reported.

**Methods:**

a retrospective study was conducted to review clinical characteristics and treatment outcomes of patients with acromegaly treated in King Chulalongkorn Memorial Hospital, Bangkok, Thailand, between 2006 and 2018.

**Results:**

eighty-four patients (31 males and 53 females) were reviewed, mean age at diagnosis was 45.7 ± 12.6 years (±SD), mean time of disease onset was 7.6 ± 6.4 years and mean follow-up period was 7.8 ± 5.3 years. The most common presenting symptoms were maxillofacial change (96.8%) and acral enlargement (94.7%). Hypertension (39.3%), diabetes mellitus (28.6%) and dyslipidemia (23.8%) were prevalent co-existing conditions. Four patients were identified having cancer at presentation; however, no additional malignancy was reported during the follow up. Most patients harbored macroadenomas, only 10 were found to have microadenomas. The outcomes of treatment were controlled disease in 70% of microadenoma and 64.9% of macroadenoma. Permanent loss of pituitary function was found in about 21.3% and there was one case reported of mortality. The logistic regression analysis for controlled disease outcome showed the IGF-I index after surgery was associated with controlled disease outcome with statistically significant result (P-value=0.006).

**Conclusion:**

our study offers descriptive clinical data of case series of acromegalic patients, which had favorable outcomes comparable with previous reports. In addition, IGF-I index after surgery is a predictive parameter for outcome of treatment.

## Introduction

Acromegaly is a rare disease with distinctive clinical manifestations. The pathogenesis of the disease are the excess of insulin-like growth factor (IGF)-I and growth hormone (GH) levels usually arising from pituitary somatotroph adenoma. Its annual incidence is 4-5 cases-million with a prevalence of 28-137 cases-million [1]. Affected patients encountered 2-2.5 times higher mortality rate compared to general populations based on cardiovascular risks assembling in acromegalic patients such as diabetes mellitus, dyslipidemia and hypertension [2]. Furthermore, acromegaly is associated with an increased risk of cancer, cardiomyopathy and sleep apnea. Transsphenoidal surgery (TSS) is the preferred treatment for most patients. Radiotherapy and medical treatment with somatostatin analogs are included to achieve clinical improvement and control the disease. Although its clinical characteristics are prominent, the slow progression of the disease causes gradual changes and delay in diagnosis. Nowadays, there are few data available on acromegalic patients in Thailand. In this study, we aimed to review clinical presentations and treatment outcomes of acromegalic patients treated at our center throughout the past decade.

## Methods

**Study design and data collection** this retrospective study reviewed acromegalic patients treated and followed up at King Chulalongkorn Memorial Hospital, a tertiary referral center in Bangkok, Thailand, between 2006 and 2018. Data from all patients diagnosed with acromegaly and coded under ICD-9 and 10; E22.0, were extracted from an electronic database. Inclusion criteria were patients aged more than 15 years and biochemical confirmation of acromegaly starting from elevated IGF-I levels for age [3] and non-suppressed nadir GH levels (>1 ng/mL) after a 75-g oral glucose tolerance test (OGTT) [2,4]. Clinical characters at baseline included clinical presentations, co-morbidities, family history, laboratory results and imaging results. The duration of onset was estimated as the time interval between the clinical onset of disease and the time when the diagnosis was performed. The duration of follow-up was estimated as the time between the first and last GH/IGF-I status assessment. This study explored all employed treatment modalities; surgery, conventional fractionated radiotherapy, medical treatments including dopamine agonists (DAs) or somatostatin analogs (SSAs). Data collection for clinical outcomes included treatment outcomes, pathological results, clinical improvements, laboratory results during follow-up, complications after treatment, patient morbidities such as hypopituitarism, colonic polyp/cancer or other malignancies and also death.

**Laboratory measurements:** GH and cortisol assays were measured by immunochemiluminescent assay (ICMA, IMMULITE 1000 systems). The measure for IGF-I, free thyroxine (FT_4_), thyroid stimulating hormone (TSH), adrenocorticotropic hormone (ACTH), follicle stimulating hormone (FSH), luteinizing hormone (LH), testosterone (T) and estradiol (E_2_) were performed by ICMA (Roche Cobas E601, USA). Serum prolactin (PRL) was measured by a chemiluminescent microparticle immunoassay (CMIA) (Abbott Laboratories, USA). The analytical sensitivity for GH and IGF-I assays are 0.01 ng/mL and 15 ng/mL respectively. IGF-I index was calculated by dividing IGF-I levels by the age-adjusted upper limit of normal levels [5]. A morning cortisol <3 μg/dL or a peak response to dynamic stimulation with 250μg of synthetic ACTH (Cortrosyn) or insulin-induced hypoglycemia <18 μg/dL was considered as evidence of hypocortisolism. Secondary hypothyroidism was defined as a FT_4_<0.8 ng/dL with an inappropriate low TSH response. Serum T was measured in male patients before surgery and at follow-up. Secondary hypogonadism was defined as a T level <300 ng/dL in males and amenorrhea in females with inappropriately low FSH and LH levels. Imaging data were studied by maximum diameter and invasion of tumor from magnetic resonance imaging (MRI) study. Tumor size was classified as microadenoma (<1 cm) and macroadenoma (≤1 cm). An invasive tumor was defined as tumor that extended beyond its capsule and invaded contiguous structures.

**Treatment outcome groups:** the treatment outcomes were categorized into 2 groups; controlled disease group defined by achieving biochemical remission criteria (normalized IGF-I levels for age and nadir GH levels ≤1 ng/mL after an OGTT) including ones with discordant GH and IGF-I level. Persistent disease group defined by not achieving biochemical remission both of IGF-I and GH level criteria. Hormonal discordant results were either normalized IGF-I levels with non-suppressible GH levels or elevated IGF-I level with nadir GH ≤1 ng/mL after an OGTT. In our center, administration of intravenous hydrocortisone was applied during surgical operation in all cases. Post-operatively, morning cortisol was assessed at 12-24 hours after discontinuing the hydrocortisone. Clinical assessment and evaluation of pituitary functions at 3, 6 and 12 months after surgery and annually thereafter. MRI was repeated at 6-12 months according to the disease status.

**Statistical analysis:** continuous variables were described by mean with standard deviation (SD), and were compared by using student t-test. For categorical variables, the results were reported by count with percentages and were compared by using chi-square test. Logistic regression analysis was used to determine clinical variables in association with controlled disease outcome. A two-tailed P-values less than 0.05 were considered statistically significant. All analyses were performed by using STATA version 16.0.

**Ethical considerations:** the study was approved by the Ethics Committee of Faculty of Medicine, Chulalongkorn University, in compliance with the international guidelines for human research protection as Declaration of Helsinki and International Conference on Harmonization in Good Clinical Practice (ICH-GCP).

## Results

**Patients:** one hundred consecutive patients with a diagnosis of acromegaly were extracted from an electronic database. Four cases were excluded due to not meeting diagnostic criteria of acromegaly. Twelve cases with incomplete data were excluded. A total of 84 patients, 55 females and 29 males, were included in the analysis. The mean duration of follow-up was 7.8 ± 5.3 years (range 1-28 years) (Table 1). The mean age at diagnosis was 45.7 ± 12.6 years (range 17-76 years) with mean duration of onset of 7.6 ± 6.4 years (range 3 month-35 year). The age at diagnosis was earlier in male at 45.5 ± 13.6 years than of female at 47.1 ± 12.7 years.

**Table 1:** clinical characteristics of patients categorized by treatment outcome groups (controlled disease group, N=55 and persistent disease group, N=29)

Characteristics	Total (N=84)	Controlled disease group (N=55)	Persistent disease group (N=29)	P-value
Female; n (%)	53 (63.1)	37/55 (67.3)	16/29 (55.2)	0.275
Age at diagnosis, years^‡^	45.7 ± 12.6	44.8 ± 13.1	47.2 ± 11.6	0.405
BMI, kg/m^2‡^	27.4 ± 4.3	27.1 ± 4.3	28.0 ± 3.7	0.463
Onset, years^‡^	7.6 ± 6.4	7.8 ± 6.0	7.4 ± 7.2	0.816
Hypertension, n (%)	33 (39.3)	21 (38.2)	12 (41.4)	0.775
Diabetes mellitus, n (%)	24 (28.6)	15 (27.3)	9 (31.3)	0.717
Dyslipidemia, n (%)	20 (23.8)	7 (24.1)	13 (23.6)	0.959
Sleep apnea, n (%)	14 (16.7)	8 (14.6)	6 (20.7)	0.473
Cardiomegaly, n (%)	12 (14.3)	7 (12.7)	5 (17.2)	0.574
GH level, ng/mL^‡^	29.2 ± 30.3	25.5 ± 33.6	36.43 ± 21.8	0.162
Baseline IGF-I level, ng/mL^‡^	652.5 ± 220.3	593.9 ± 222.9	753.3 ± 178.2	0.003
Baseline IGF-I index^‡^	3.1 ± 1.1	2.9 ± 1.0	3.5 ± 1.1	0.016
Hyperprolactinemia, n (%)	21 (28.4)	11 (22.9)	10 (38.5)	0.157
Hypogonadism, n (%)	25 (40.3)	16 (39.0)	9 (42.9)	0.771
Hypocortisolism, n (%)	5 (6.3)	3 (6.3)	2 (8.3)	0.743
Hypothyroidism, n (%)	1 (1.3)	1 (1.9)	0	0.298
Tumor size, mm^‡^	20.7 ± 9.8	19.5 ± 8.7	23.1 ± 11.3	0.112
Macroadenoma, n (%)	74 (88.1)	48 (87.3)	26 (89.7)	0.749
Invasive tumor, n (%)	44 (61.9)	31 (64.6)	13 (56.5)	0.513
IGF-I levels after surgery, ng/mL^‡^	353.9 ± 199.8	291.3 ± 175.2	468.1 ± 194.4	0.001
IGF-I index after surgery^‡^	1.7 ± 0.9	1.3 ± 0.7	2.2 ± 0.9	0.001
Follow up duration, years^‡^	7.8 ± 5.3	8.1 ± 4.8	7.2 ± 6.2	0.477

IGF-I index was calculated from IGF-I level divided by age-adjusted upper limit of normal IGF-I level; in cases with multiple times of surgery, the IGF-I index after surgery was the value after first surgery only; ^‡^results presented in mean ± standard deviation (SD)

**Clinical features at the presentation:** maxillofacial change 96.8% and acral enlargement 94.7% were the two most frequent characteristics found in all patients. Snoring (46.3%) and thyroid gland enlargement (48.9%) were found in about half of the patients. Around one-third experienced musculoskeletal problems (37.2%) and headache symptoms (36.2%). The most common presenting symptoms bringing patients to seek advice were maxillofacial change (34%), headache (18.1%) and acral enlargement (14.9%). Two cases had a pituitary apoplexy. Vision was affected about one-third of the patients, bitemporal hemianopia is the majority of these cases. Common co-morbidities were hypertension (39.3%), diabetes (28.6%), dyslipidemia (23.8%), sleep apnea (16.7%) and cardiomegaly (14.3%). Screening for malignancies were carried out and found that 15 cases had colonic polyps, 2 cases (5.1%) had colorectal cancer and 2 cases had well-differentiated thyroid carcinoma. All patients with malignancy were female with aged over 50 years. Genetic syndrome with acromegaly was found in one case of McCune-Albright syndrome: a 21-year-old man presenting with tall stature, asymmetrical face and café-au-lait spot was found to harbor a large invasive pituitary tumor [6].

**Hormonal evaluation and imaging studies:** at baseline, mean GH levels were 29.2 ± 30.3 ng/mL, mean IGF-I levels were 652.5 ± 220.3 ng/mL and IGF-I index were 3.1 ± 1.1. Hyperprolactinemia was encountered in 28.4% of all patients. Hypogonadism was found in 40.3% and hypocortisolism was 6.3%. Thyroid status was mostly normal, except for one case who had acromegaly with co-secreted thyrotropin-producing adenoma (TSHoma) exhibiting hyperthyroidism [7] and one case for secondary hypothyroidism. Among 84 cases, macroadenomas were predominant, with only 10 identified microadenoma cases. No empty sella was noted. Mean maximum diameter of tumors were 20.7 ± 9.8 mm, range: 4-50 mm, and invasive tumor were found in 44 cases (61.9%) of all patients. Pathological reviews categorized by immunohistochemistry staining showed GH producing pituitary tumor as the most prevalent result at 55.2%, followed by GH with PRL co-producing tumor at 26.4%, and GH + PRL + FSH/LH producing tumor at 18.4%.

**Treatment modalities and outcomes:** the mainstay treatment modality was transsphenoidal surgery (TSS), which was conducted in 81 patients. The other three cases (microadenoma 1 case and macroadenoma 2 cases) denied surgery due to their old age and were treated with radiation only. Patients who had multiple surgical treatment were about 22/81 (27.2%), with three cases reporting a maximum of four surgical experiences. There were seventeen patients who had combined radiotherapy (20.2%), seven patients with combined medical treatment (8.3%) and three patients who had multimodality approaches with surgical, medical and radiation treatment (3.6%) (Table 2). Two patients were treated pre-operatively with SSAs to improve their conditions: heart failure and thyrotoxicosis. Two patients were treated with DA post-operatively according to their co-secretion with PRL hormone confirmed with positive IH staining for GH and PRL.

**Table 2:** treatment modalities used and outcomes according to microadenoma and macroadenoma

Treatment modalities	Microadenoma (n=10)		Macroadenoma (n=74)		All n (%)
	Controlled (n=7)	Persistent (n=3)	Controlled (n=48)	Persistent (n=26)	
Surgery only	6	2	30	16^a^	54 (64.2)
Surgery/radiotherapy	0	0	12	5	17 (20.2)
Surgery/medication	1	0	3	3	7 (8.3)
Surgery/medication/radiotherapy	0	0	3	0	3 (3.6)
Radiation only	0	1	0	2	3 (3.6)
All, n (%)	7 (70%)	3 (30%)	48 (64.9%)	26 (35.1%)	

a Death 1 case

We identified 14 cases (2 microadenoma, 12 macroadenoma) out of 84 cases meeting the early remission criteria within twelve months after surgical treatment. There were about 46.8% and 12.5% suppressible GH levels after OGTT performed within 1 year after first surgical treatment in controlled disease and persistent disease groups, respectively. In addition, none of the patients in persistent disease group had normalized IGF-I level within 6 months after surgery. During entire follow up period, the controlled disease outcomes were accomplished in 55 cases: 7 microadenomas and 48 macroadenomas, accounting for 65.4% of total 84 cases. Among 55 cases, there were 20 cases with discordant hormonal results; 9 cases of normal IGF-I levels with non-suppressible nadir GH levels after OGTT, and 11 cases of elevated IGF-I levels with controlled nadir GH levels. There were 29 cases who remained persistent disease: 3 microadenomas and 26 macroadenomas. No recurrent case reported during the follow-up.

**Pituitary reserved function and clinical improvement after surgery:** post-operative complication evaluation showed 21.7% of transient diabetes insipidus (DI). Permanent hormonal deficiency findings were hypocortisolism 24.1%, hypothyroidism 17.5%, hypogonadism 14.9% and panhypopituitarism 21.3%. No meningitis, CSF leaks or other serious adverse outcomes were detected during admission for operation, with the exception of one patient who had major blood loss intra-operation and subsequently died from severe hospital acquired infection. However, no additional death reported during the follow-up. Clinical and laboratory improvement at last follow-up period were analyzed showing soft tissue improvement of 57.7% and visual field recovery of 42.3%. Remission of hypertension and diabetes mellitus was 9.1% and 33.3%, respectively.

**Predictive factors associated with treatment outcome:** the logistic regression analysis of controlled disease outcome for each clinical variable demonstrated in Table 3. Both of IGF-I index at baseline and within 6 months after surgery were inversely associated with the likelihood of achieving controlled disease outcome, with statistical significance P-value 0.025 and 0.002 for baseline IGF-I index and after surgery respectively. In multivariate logistic regression analysis, we adjusted for only variables with significant results from univariate analysis. From the study, we can assume that each one time higher of IGF-I index after surgery was associated with 71% lower chances to achieve controlled disease outcome (adjusted OR 0.29, 95%CI 0.12 - 0.71, P-value=0.006).

**Table 3:** logistic regression analysis results for clinical variables in association to controlled disease outcome

Clinical variables	Univariate analysis		Multivariate analysis	
	Crude OR (95%CI)	P-value	Adjusted OR (95%CI)	P-value
Female gender	0.60 (0.24 - 1.51)	0.276	-^†^	
GH level, ng/mL	0.99 (0.97 - 1.01)	0.195		
Tumor size (mm)	0.96 (0.92 - 1.01)	0.116		
Baseline IGF-I index	0.55 (0.33 - 0.93)	0.025^Š^	0.73 (0.39 - 1.37)	0.329
IGF-I index after surgery	0.26 (0.11 - 0.61)	0.002^Š^	0.29 (0.12 - 0.71)	0.006^Š^

† non-significant results from univariate analysis were not included in the multivariate analysis model; ^Š^ results with statistical significance, (P<0.05)

## Discussion

We represent comprehensive retrospective data of patients with acromegaly of our center. It delineates the details of clinical presentations, patient characteristics and treatment outcomes in a referral pituitary center in Thailand. Our study revealed a slight female predominance; however, the age at diagnosis was earlier in male than female [8,9]. As its subtle progression, the signs and symptoms of the disease emerged nearly 8 years earlier to establish the diagnosis. Moreover, the long delay to diagnosis more than 10 years was observed in 31 cases in our series. Our patient characteristics and clinical presentations also shared similarities with other studies in other different ethnicities and populations [9-11]. The leading presentation was maxillofacial change which was slightly more observable than acral enlargement. According to the fact that Thai people do not usually wear rings and prefer sandals than casual shoes, enlarged hands and feet were clinically unnoticed. Prevalence of other common co-morbidities including diabetes mellitus, hypertension and sleep apnea were comparable as described in the study [2]. Nevertheless, fewer cancers with only four cases were found. Slightly higher numbers of macroadenoma reaching 90% of all cases in our series, which differs from the data from other studies that macroadenoma is about 70-80% of the patients [9-12]. Besides, nearly two-thirds of all cases were invasive tumors.

Treatment approaches in our series were surgery alone in two-thirds of the cases and combined with either radiation or medication in about one-third of all cases. The treatment outcomes in our study were comparable with previous reviews using the same remission criteria, 70% in microadenomas and 64.9% in macroadenomas [10]. Articles published after the year 2000 showed various remission outcomes that used the same remission criteria as our study including reported ranges of 22% in Italy [9], 41% in Greece [10], 89%, 79% for microadenoma and macroadenoma, respectively in Japan [11] and 86.7%, 72.3% for microadenoma and macroadenoma, respectively in a Korean study [12].

The recent study showed the baseline characteristics that affected the treatment outcome, which were male gender, older age, smaller tumor size and lower baseline IGF-I index [5]. In our study reviewed baseline IGF-I index and IGF-I index post-operatively within 6 months were associated with treatment outcome. These reflected the great need and impact of early diagnosis to be a key success factor for curative outcome. There was less medication therapy with long acting SSAs observed in our study, only nine cases who underwent combined medication treatment and two cases pre-operatively. In our reviews, it supported good results as well which had received medication in controlled disease condition with mild adverse effects including bloating and pain at the injection site. Combined treatment with DA (bromocriptine) in patients with co-secreted GH and PRL tumor also gets a good result, all them were in controlled group and no side effect from medication.

The limitations of our study include its retrospective study, high incomplete data from the loss to follow-up (12.5%) and performed in a single center. Information about cardiac co-morbidities including valvular diseases and cardiomyopathy was not completely evaluated pre-and post-operatively.

## Conclusion

This study provides the inclusive review of acromegalic incidents, clinical presentations and outcomes treated in a referral center in Thailand. It shows favorable results of patient outcomes, with only few unfavorable complications demonstrated during the treatments. These results will reflect the work of our pituitary care team in the past and offer more effective manners of improvement in the future. In addition, post-operative assessment with IGF-I index may be a predictive outcome of the treatment.

### What is known about this topic

- Clinical manifestation of acromegaly is distinctive, yet there remains a delay in diagnosis as also seen in this review (7.6 ± 6.4 year);
- Hypertension, diabetes and dyslipidemia are common comorbidities in acromegaly, accounted for one-third from this study.

### What this study adds

- IGF-I index prior and after surgery are both significant parameters associated with treatment outcome.
